# Consumers’ Attitudes Facing Entomophagy: Polish Case Perspectives

**DOI:** 10.3390/ijerph17072427

**Published:** 2020-04-02

**Authors:** Agnieszka Orkusz, Wioletta Wolańska, Joanna Harasym, Arkadiusz Piwowar, Magdalena Kapelko

**Affiliations:** 1 Department of Biotechnology and Food Analysis, Wroclaw University of Economics and Business, 53-345 Wroclaw, Poland; joanna.harasym@ue.wroc.pl; 2 Department of Forecasts and Economic Analysis, Wroclaw University of Economics and Business, 53-345 Wroclaw, Poland; wioletta.wolanska@ue.wroc.pl; 3 Department of Economics and Organization of Food Economy, Wroclaw University of Economics and Business, 53-345 Wroclaw, Poland; arkadiusz.piwowar@ue.wroc.pl; 4 Department of Logistics, Wroclaw University of Economics and Business, 53-345 Wroclaw, Poland; magdalena.kapelko@ue.wroc.pl

**Keywords:** environmental concern, edible insects, food neophobia, behavior change

## Abstract

Based on high nutritional value and low production costs, edible insects are an excellent and sustainable source of animal proteins. However, completely replacing meat with edible insects requires a change in consumer mentality not only in Poland, but also in other European countries. In western countries, most people reject eating insects, mainly for cultural reasons. Concerning this, the objective of the study was to examine the knowledge, behavior, and attitudes of the Polish community about edible insects and to understand the main factors driving edible insect consumption. The study was held at the Wroclaw University of Economics and Business, Poland and consisted of two parts: The survey (among 464 students) and the tasting session (among 402 participants). The main findings suggest that there is low willingness to adopt edible insects as a meat substitute among Polish students due to the psychological barriers, such as neophobia and disgust. However, the willingness to eat processed insect food (bread, biscuit) is far higher than for unprocessed whole insects. Environmental benefits are the factors that least affected students’ willingness to try edible insects. Additionally, the tasting session of the bread with powdered insects was attended by the vast majority of participants, which indicates that a positive sensory experience can improve the acceptability of insects as food.

## 1. Introduction

The growth of the world’s population, projected to soar to a staggering 9.6 billion by 2050, will inevitably result in an increased demand for food, namely animal proteins. Animal proteins account for about 40% of global protein intake, but their production is highly inefficient, and about 2–15 kg of plant material is needed for 1 kg of animal products. In addition, livestock farming and meat consumption are associated with high environmental impacts and the release of greenhouse gases, making these practices unsustainable [1]. A particularly interesting alternative to animal proteins for human consumption and production of animal feed is seen in insects, algae, and meat obtained from in vitro cultivation. The intake of insects, known as entomophagy, is a foreign concept to western culture, however, it is an integral part of the diet of at least 2 billion people in Asia, Latin America, Africa, and Australia [2,3,4,5]. Some of the most commonly consumed species are beetles (Coleoptera; 31%); caterpillars (Lepidoptera; 18%); and bees, wasps, ants (Hymenoptera; 14%). They are followed by grasshoppers, locusts, and crickets (Orthoptera; 13%); cicadas, grasshoppers, meal worms (Hemiptera; 10%); termites (Isoptera) and dragonflies (Odonata; 3%); flies (2 %); and other genus (5%) [6]. 

In recent years, many products called “super foods” have appeared in the sector of the, so called, healthy food market. This specific group of products also includes edible insects, rich in easily digestible protein, unsaturated fatty acids, iron, and vitamin B_12_, at levels which are not available in any plant food [3,7,8,9]. Schmidt et al. (2019) [10] found that levels of vitamin B_12_ were 1.08 µg/100 g for mealworm, 2.88 µg/100 g for cricket, 0.84 µg/100 g for grasshopper, and 13.2 µg/100 g for dry weight cockroach. 

Edible insects are rich in essential nutrients, but their nutritional value varies greatly depending on the species, life stage (higher protein content of adults compared to stages of development between successive larval linings), habitat, and the diet of insects [2]. Depending on the species, the protein content in various edible insects ranges from 13%–81% [6], which usually exceeds beef and poultry as well as fish and seafood, which are in range of 19–26% and 13–28% of protein, respectively [11]. Edible insects can also be beneficial as a supplement to the plant diet based on cereals due to their high content of exogenous amino acids.

From the environmental costs point of view, it is difficult to find a more environmentally friendly food production model than that of edible insects [12,13]. Insects effectively absorb nutrients from feed; from the same amount of dry food, insects are able to produce several times more of mass than poultry or cattle. Moreover, they are generally satisfied with food, which from the agricultural point of view is a waste; leaves, bran, straw, vegetable, and fruit pressing marc. Water consumption for insect cultivation is several hundred times lower than for crops, cattle, pigs, or poultry, and greenhouse gas emissions are also lower [6]. A negligible amount of excrement produced by insects can be utilized as an ecological fertilizer. Insect breeding can be intensive; it takes up little space, in addition, it can be any space, without the need to use huge areas of land for pasture or arable land. Insects are cold-blooded animals, therefore their killing can be carried out in a humane way, because it is enough to cool them, without pain, suffering, and stress. An undoubted economic aspect is the fact that insect production is cheap. Producing any other food with the same nutritional value as insects requires a lot more investment. It is well known that insect breeding does not need expensive technologies or large amounts of work by a qualified workforce. 

The introduction of insects to the human diet has recently gained huge attention. The overview of the key quantitative studies on the perception and acceptance of insects as food in Europe (in Belgium, Germany, Hungary, Italy, Netherlands, and Switzerland) was prepared by Hartmann and Siegrist (2017) [14]. However, there is a lack of research on the psychological mechanisms of acceptance/rejection of food containing edible insects. Such research would lead to a deeper understanding of this phenomenon and proposing an approach to changing eating habits and introducing other types of food to mass production. A change in attitudes towards such foods may prove necessary in the short or long term to ensure a healthy diet for entire human populations. An acceptance of a new food largely depends on the level of food neophobia, i.e., how strong the fear of new food is characteristic of the human psyche. People with a lower level of food neophobia consume a wider range of foods compared to people with higher levels of food neophobia [15]. 

Due to the growing interest in entomophagy in western countries, the first purpose of this study was to examine (by questionnaire) the knowledge, behavior, and attitudes of students about edible insects and to understand the main factors of edible insect consumption. The level of food neophobia among Polish students was also determined. The relationship between personal variables such as sex, place of residence, monthly income, travel destinations, protein source in the diet, and food neophobia levels was assessed as well. The second objective of the study was to evaluate the willingness of the Polish community to try foods containing insects. This was achieved during the tasting session by preparing bread based on edible insects. The results of the study can help to understand whether Polish consumers are ready to accept insects in their diet and can be used by the food industry stakeholders to develop production and/or marketing strategies that will increase the acceptance of insects as food. The third objective was to statistically model the main factors determining the acceptance of edible insects in the students’ diet.

The paper is organized as follows: Section 2 introduces the study design and modeling approach; Section 3 presents the results of the questionnaire, statistical modeling, and tasting session; while Section 4 provides the results discussion; and Section 5 presents conclusions.

## 2. Materials and Methods 

### 2.1. Research Framework

**Figure 1:**
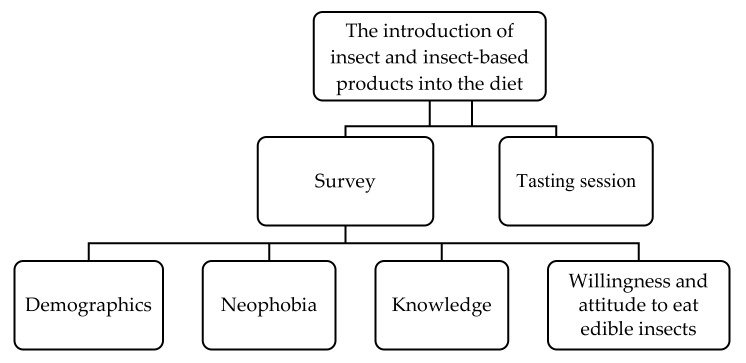
Study design.

The survey and the tasting session were conducted (Figure 1) at the Wroclaw University of Economics and Business (Poland) in June 2019 and in September 2019, respectively. 

#### 2.1.1. Participants

In total, 866 participants took part in the study, including 464 that completed the survey, and 402 that had the opportunity to taste wheat bread with a 20% addition of powder from cricket (Acheta domesticus) during a tasting session.

The survey was held among students aged between 18 and 24 years old. The participants were randomly recruited from students who have applied voluntarily to participate in the survey on the campus of the Wroclaw University of Economics and Business in Wroclaw. There were 464 questionnaires distributed, of which 454 were correctly completed. 

The tasting session was organized as a part of the event entitled “Edible insects in human nutrition” during the Lower Silesian Science Festival in September 2019. Admission to the event was free of charge, voluntary, and open to all people from outside the university community. A total of 402 participants aged 18–78 took part in the event. 

#### 2.1.2. Survey

The survey consisted of questions regarding demographics, knowledge, and willingness to consume edible insects that were based on previous studies [16]. The survey consisted of four parts. The first part presented consumer demographics: Sex, place of residence, household income, travel destination, and protein source in the diet (Table 1). 

The second part comprised the Food Neophobia Scale (FNS) according by Pliner and Hobden (1992) [17]. The FNS consists of ten statements, five positively worded (I am constantly sampling new and different foods; I like foods from different countries; At dinner parties, I will try a new food; I will eat almost anything; I like to try new ethnic restaurants) and five negatively worded (I don’t trust new foods; If I don’t know what is in a food, I won’t try it; Ethnic food looks too weird to eat; I am afraid to eat things I have never had before; I am very particular about the foods I will eat) rated on a five-point Likert scale (with endpoints: ‘definitely disagree’ and ‘definitely agree’). Total FNS results are the measure of food neophobia, i.e., unwillingness to consume new or novel foods [17]. In order to determine nutritional neophobia, the results were divided into three groups: Low, medium, and high level of neophobia. A higher the number of points obtained indicated a higher the level of neophobia in the studied group. This type of classification has also been used in previous studies [17,18,19]. 

In the third part, participants were asked whether they had previously heard of: Edible insects, eating insects in other cultures, restaurants serving edible insects, and whether they had known that edible insects are a source of protein. 

In the fourth part, participants responded (yes/no/I don’t know) to a series of statements to indicate: Consumer willingness to try edible insects and insect-based products (bread, cake) in diet and in animal nutrition; preferred form of insects (whole insects prepared alone, whole insects with the addition of spices or covered with chocolate, insects as insect flour to make: biscuits, bread, burger, pate); and consumer attitudes towards the benefits of eating insects. Students were also asked to answer another question (if they had previously eaten insects, if insect eating is disgusting and whether the idea of eating insects makes them feel sick, would they use edible insects if they knew that: insects are easy to prepare, do not require long heat treatment, they can be bought near the place of residence, they are easily available in stores and supermarkets). In the last question respondents were asked about factors (price, nutritional value, food safety, quality, taste, appearance, and environmental benefits) influencing consumer willingness to try eating insects. Students were asked to choose only the four most important factors out of seven and to give them points from 1 to 4, where “1” was the least important, and “4” was the most important. Respondents took approximately 15 min to complete the survey and data were collected within a three-week period. Participation was voluntary.

For the analysis of statistical material, cross tables were determined and the chi-square (χ^2^) independence test was used. These statistical analyses were performed using the Statistica software programme, version 13.0 (TIBCO Software Inc., Palo Alto, CA, USA). Results with *p* < 0.05 were considered statistically significant. 

#### 2.1.3. Statistical Modeling

Because the acceptance of edible insects (“yes”, “I don’t know”, “no”) was a qualitative variable to indicate the main factors that determine it, the logistic regression model was applied to measure it.

In the course of the further analysis, the respondents were divided into two groups depending on the answers to the survey question they gave: “Would you accept edible insects in your diet?”. The first group consisted of those that would accept edible insects in their diet (81 people), and the second group consisted of those that would not accepted edible insects or do not have an opinion (373 people). Assuming that the propensity to accept edible insects in the diet is a qualitative variable with two states, a binomial logistic regression model was used to model it.

Logistic regression models are used to explain the qualitative variable Y depending on the level of exogenous variables X_1_, X_2_, ..., X_k_ (qualitative or quantitative). The binomial logistic regression model (logit model) is intended to explain a dichotomous explained variable, usually represented by a zero–one variable. The logit model is a special case of the generalized linear model:

(1) $$g\left(\mu \right)=\beta_{0}+\beta_{1\;}X_{1}+\ldots +\beta_{k}X_{k}$$

where *β*_0_ is a free word*, β*_1_, ..., *β*_k_ are regression coefficients, and g is a binding function determining the relationship of the average value of the explained variable

$$\mu =E\left(YIX_{1}=x_{1},\;\ldots ,\;X_{k}=x_{k}\right)$$

with a linear predictive function.

In the logit model$\;\mu =p=P\left(Y=1IX_{1}=x_{1},\;\ldots ,\;X_{k}=x_{k}\right)$, and the binding function called logit has a form:

(2) $$g\left(p\right)=logit\left(p\right)=\ln\left(\frac{p}{1-p}\right).$$

Finally, the logit model takes the form:

(3) $$p=P\left(Y=1IX_{1}=x_{1},\;\ldots ,\;X_{k}=x_{k}\right)=\frac{\exp(\beta_{0}+\sum_{i=1}^{k}\beta_{i\;}x_{i})}{1+\exp(\beta_{0}+\sum_{i=1}^{k}\beta_{i}x_{i})}$$

In the model, the explained variable Y was the binary variable: the propensity to accept edible insects in the diet:

$$Y=\left\{\begin{array}{c} 1-acceptance\;of\;edible\;insects\;in\;the\;diet,\;\\ 0-\;no\;acceptance\;of\;edible\;insects\;in\;the\;diet\;or\;no\;opinion.\; \end{array}\right.$$

The potential explanatory variables of the model were: Sex (X_1_; variable with a value of 1 in men and 0 in women); level of neophobia (X_2_; variable of 1 for low neophobia and 0 for medium and high neophobia level); place of residence (X_3_; variable of 1 for city dwellers over 20,000 and 0 for other places of residence); monthly income (X_4_; variable of 1 for a budget bigger than 1000.01 PL per person and 0 for a budget not exceeding 1000.00 PL); travel to Europe (X_5_; variable of 1 for travel to Europe and 0 for otherwise); travel to Asia (X_6_; variable of 1 for travel to Asia and 0 for otherwise); travel to Africa (X_7_; variable of 1 for travel to Africa and 0 for otherwise); and travel to North and South America (X_8_; variable of 1 for travel to North and South America and 0 for otherwise).

Regression coefficients were estimated using maximum likelihood estimation and are presented as odds ratios. In order to verify the model correctness of the likelihood ratio test, Hosmer–Lemeshow test, the percent of correct predictions and Nagelkerke’s R^2^ were determined.

The model analysis was carried out in the PS IMAGO PRO version 5.1 computer package (Predictive Solutions Sp. z o.o., Kraków, Poland) based on a sample of 454 observations. 

#### 2.1.4. Tasting Session

**Figure 2:**
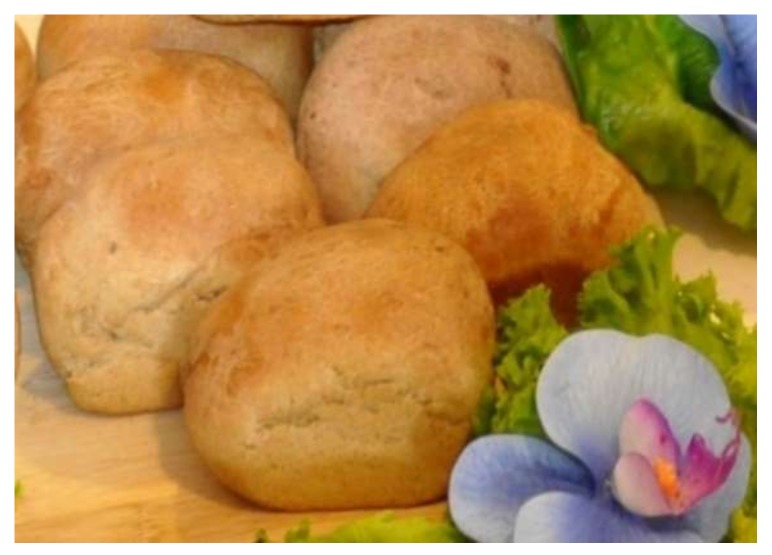
Bread loaves with insect powder addition.

**Figure 3:**
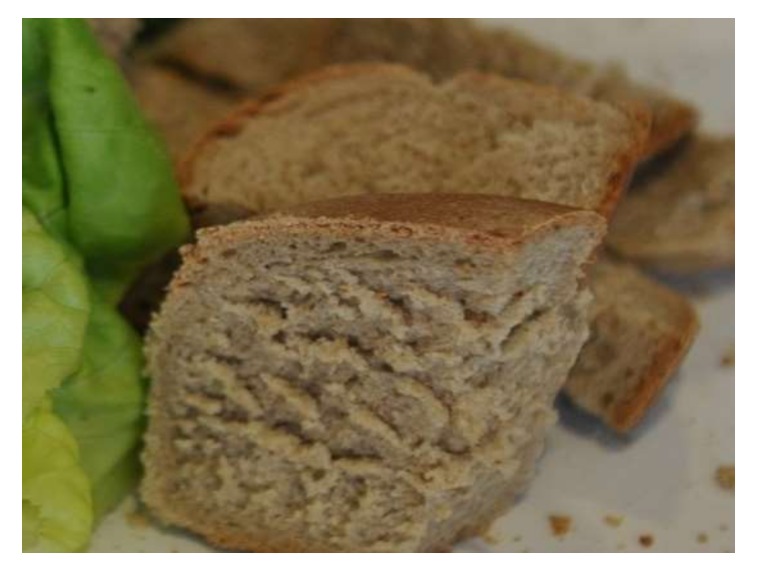
Piece of bread with insect powder addition.

Before tasting, participants were informed about the type of insects added to the bread, as well as the safety of bread preparations and the allergenic potential of insects. A total of 56 loaves of bread were prepared for tasting, all with dimensions 9 cm × 5.5 cm × 5 cm. Ten loaves of bread were presented in full and 46 were cut. Bread was presented on beige paper trays, surrounded by lettuce and flowers (Figure 2 and Figure 3). 

#### 2.1.5. Ethical Statement

All subjects gave their informed consent for inclusion before they participated in the study. The study was conducted in accordance with the Declaration of Helsinki, and the protocol was approved by the Research Ethics Committee of Adaptive Food System Accelerator, Research Centre of Wrocław University of Economics (CREABIOECON/1/2019).

## 3. Results

### 3.1. Survey

#### 3.1.1. Consumer Demographics

A significantly higher proportion of participants were women (64.8%; Table 1). The size of the respondents’ place of residence varied. Among all the surveyed, 125 people (27.5%) lived in the countryside and 152 people (33.5%) in a large city with over 500,000 inhabitants. In the studied group, consumers with the lowest and highest monthly income constituted 24.2% and 25.1%, respectively. The students mostly traveled around Europe (97.8%). Asia and South America, continents where insects have been consumed for centuries, were visited by 10.4% and 2.6% of participants, respectively. 

Considering the delivery potential of edible insects as an alternative source of protein for meat/fish/seafood, consumers’ protein consumption was included as an additional demographic characteristic. The vast majority of respondents consumed meat (96.9%), mainly poultry (91.4%), which is a source of wholesome animal protein. The responses indicated that other types of protein sources were consumed by over 24% of consumers (except for seafood). Importantly, the diet of 198 respondents included fish, which is a good source of animal protein containing small amounts of fat rich in saturated fatty acids. Only 14 consumers indicated they did not eat meat, fish, or sea food (Table 1). 

#### 3.1.2. Food Neophobia

Most of the respondents (64.8%) had an average level of neophobia (Table 2). A low level of neophobia was found by 16.7% of the surveyed people, while 18.5% of the students showed a high level of unwillingness to try new or unknown food. Nearly twice as many men (23.8%) than women (12.9%) represented a low level of neophobia. Sex was significantly associated with the level of food neophobia. More women (21.8%) than men (12.5%) had a high neophobic attitude towards novel foods. Neophobia was not influenced by the place of residence or income (Table 2). It was shown, however, that the neophobia level of the participants was related to the destination of travel and the source of protein in the diet (Table 2). People who traveled to Asia as well as North and South America had a lower level of food neophobia.

Due to the fact that only 2.2% of the respondents did not travel to any European country, and 3.1% indicated “none of the above” as a response about the sources of protein in their diet (Table 1), the impact of these features on the level of neophobia was not studied.

#### 3.1.3. Knowledge 

Table 3 presents the distribution of answers to the survey questions and *p*-values for the X^2^ test for the analysis of the relationship between answers to survey questions and sex, place of residence, and income.

Most of the participants surveyed indicated that they heard about both the edible insects (88.1%) and the eating insects in other cultures (94.1%; Table 3). Respectively, 9.1% and 4.6% of the sample claimed to have never heard about edible insects and the eating of insects in other cultures. More than half of respondents indicated that they had heard of restaurants serving edible insects (54.2%) and knew that insects are a source of protein (67.2%). Sex, place of residence, and monthly income did not affect the level of knowledge in the analyzed group (Table 3; questions 1–4).

#### 3.1.4. Willingness and Attitude to Edible Insects Eating 

Although the majority of respondents had heard about edible insects (88.1%) and eating insects in other cultures (94.1%), only 7.3% declared that they had eaten insects before. In the current study, the awareness of entomophagy was not influenced by sex or place of residence. However, it was shown that people with a higher monthly income consumed edible insects more often compared to those with a lower income. 

The distribution of answers to the survey questions by sex, place of residence, and income in case of statistically significant dependences was also considered (Table S1). More women (52.38%) than men (31.88%) thought that eating insects was disgusting and made them feel sick (45.92%; 25.0%). As income increased, the percentage of people who thought that eating insects is disgusting decreased (Table S1). The same dependence was recorded for the inhabitants of the largest cities. Income did not affect the idea of being sick after eating insects among students (Table 3; questions 6, 7). Most of respondents (59.0%) indicated that they would not accept edible insects in their diet (most of them were women (67.35%)), 17.8% would accept (it is likely that they were people with low level of neophobia; Table 2), and 23.1% were undecided (Table 3; question 8). When asked if they would accept bread or biscuit from insect flour in their diet (Table 3; questions 9, 10) nearly 50% of respondents disagreed; with a significant difference between sex (more for females (54.08%; 54.42%) than males (35.0%; 36.88%)), and monthly income (the lower the income the greater the reluctance to introduce insects and insect-based products into the diet (Table S1)). The results of the study showed that consumers would be more likely to accept edible insects in the diet of animals they eat (poultry, pork, beef, fish) than in their diet. Sex and place of residence were found to have significant effects. More than 60% of respondents (more men than women) would accept insects in animal nutrition. The highest acceptance of insects in animal nutrition was found in the inhabitants of large cities. The effect of income was not significant (Table 3; question 11). Students were more willing to try whole insect with the addition of spices or covered with chocolate than the whole insects served sauté (Table 3; questions 12, 13). On the question whether consumers would try insects in non-detectable form (flour added to different products; question 14), more men than women responded yes (Table S1). At the same time, it was shown that with the increase in income, the percentage of people who were willing to try insects in invisible form increased. It is worth noting that the answers changed depending on how the question was formulated. Interesting results were obtained by comparing the answers to questions 9 and 10 (Would you accept bread/biscuit from insect flour in your diet?) with question 14 (Would you like to try insects in an invisible form—flour added to: biscuit, bread?), in which the term “invisible” was added. This resulted in a decrease in the number of people who were undecided in favor of people willing to try products (biscuit, bread) based on edible insects compared to question 9 and 10 (Table 3). More than half of those surveyed with the lowest income, more women than men, spoke negatively about the use of edible insects if they knew that: Insects are easy to prepare, do not require long heat treatment, they can be bought near the place of residence, and they are easily available in stores and supermarkets (Table 3; question 15). For more than 30% of the respondents (more often men than women), reduced food wastage, and scarcity of agricultural land positively influenced the decision to include insects in the western diets. At the same time, a high percentage of consumers (over 35%), including more women than men, were undecided (Table 3; question 16).

People participating in the study were asked to answer the question regarding which of the four factors (out of 7) are most important for them to affect their willingness to try edible insects (Table 4).

It has been shown that the taste and quality of insects had a decisive impact on the acceptance of insects as food. Significant importance was also attached to the appearance and nutritional safety of insects. Interestingly, the environmental benefits were the least influential factor in the desire to eat insects (Table 4).

#### 3.1.5. Factors Determining the Acceptance of Edible Insects in the Students’ Diet

Regression coefficients for the following variables were not significant (*p* = 0.05): The place of residence; travel to Europe and Africa; and seafood as the main source of protein in the diet. This is equivalent to the fact that the chance quotients for these variables did not differ significantly from “1”. This means that these variables did not significantly affect the acceptance of edible insects in the diet and were excluded from the set of predictors.

Table 5 presents the estimation results of the re-estimated model with a reduced number of explanatory variables. Basing on the analyses, it was found out that the factors affecting the acceptance of edible insects in the students’ diet were: sex, level of neophobia, place of residence, as well as travel to Asia and North and South America. The parameters of these variables are statistically significant for *p* < 0.033.

The equation of the model was defined as following:

(4) $$IA{\;}_{i}=-2.821+0.653S_{i}+1.452F_{i}+0.648PR_{i}+0.972TAz_{i}+1.323TAm_{i}$$

where *i* = 1, 2, 3, …, *n.*

Based on the likelihood ratio test for the analyzed model, it can be concluded that all variables were significant. The lack of significance in the Hosmer–Lemeshow test indicates the correctness of fitting the model to the predicted data. Also 84.4% of correct predictions are satisfying. While Nagelkerke’s R^2^ was not high, even for well-fit logistic regression models, it was much lower than classic R^2^ [20].

Based on the value of the odds ratios (Table 5), it was found that the factor having the greatest impact on the acceptance of edible insects in the students’ diet was food neophobia. The chance of accepting edible insects by people with low neophobia was 327% higher compared to people with medium and high neophobia. The place of travel also had a big impact on consumer preferences. Among those who traveled to North America or South America or Asia, the chance of accepting edible insects was 275% and 164% higher than for others, respectively. Sex was another important factor influencing the preferences of the respondents. The chance of insect acceptance in the diet by men was 92% higher than in case of women. Place of residence was also a factor that determined the attitudes of the respondents. The chance of eating edible insects was 91% higher among inhabitants of cities with over 20 thousand people than in other cases.

### 3.2. Tasting Session

A total of 395 people agreed to participate in the tasting session, which accounted for 98.3% of the participants. Bread was chosen as the food product as consumers were familiar with it, an attribute which has been suggested to reduce food neophobia among western consumers [21,22,23]. As the taste session was based exclusively on the desire to try food containing insects, it is not known whether the people who took part in it would include insects in their diet permanently, or whether they were just interested in what such food tastes like. This problem requires further research.

## 4. Discussion

The Food Neophobia Scale is a psychometric instrument that is used to measure food neophobia [17]. This method is used to assess individual differences in the level of nutritional neophobia in the adult population. Neophobia is the opposite of neophilia, which is expressing a general desire to try new foods [17]. According to the literature, neophobia is a genetically conditioned feature. Heredity explains 70%–78% of the variability of neophobia, but a quarter of this variance depends on environmental factors such as pre-natal nutrition, nutrition during infancy, as well as early childhood personality, eating habits, and lifestyle [17,24,25].

Many studies have shown that FNS accurately predicts responses to new products (novel foods) [15,17]. According to previous literature [21,26,27], food neophobia is a barrier for the consumption of insects for European consumers.

Based on the results of our own research, it was shown that among the analyzed demographic factors, the level of neophobia of students was influenced by sex, travel destination, and protein source in a diet. Women were more neophobic compared to men. Our results differed from that of Olabi et al. (2009) [18] and Gutiérrez-Salomón and Villanueva-Rodríguez (2016) [28] who showed that sex did not influence the level of food neophobia. They were also in contrast to Muhammad et al. (2015) [19] who found that female respondents were less neophobic compared to the male respondents. Students traveling to America and Asia, countries with a different culture and ethnic food, were less neophobic than those who did not travel to those countries. Olabi et al. (2009) [18] pointed out that number of trips taken outside the country of residence affected food neophobia level. The level of neophobia was higher for students that did not take any trips outside their country of residence. On the other hand, both the place of residence and monthly income were not significantly related to the level of food neophobia. These findings were in agreement with the work done by Olabi et al. (2009) [18] on a group of Lebanese and American college students. Different results had been reported earlier by other authors [29,30]. Tuorila et al. (2001) [29] found that the higher the level of urbanization, the lower the level of food neophobia. Also, Flight et al. (2003) [30] showed that urban Australian high school students were less food neophobic than rural adolescents. In addition, participants from rural areas showed higher level of suspicion of novel foods compared to those from urban areas [31]. Moreover, some authors even hypothesize that food neophobia may not even exist for city dwellers, but there is no such evidence in the literature [32].

According to many authors [28,29,30,33], people with higher incomes had a lower level of food neophobia compared with people with lower incomes. Flight et al. (2003) [30] concluded that exposure to a variety of cultures and higher economic status can lead to growth of knowledge of a wide range of stimuli, including food, which entails a lower level of food neophobia. Openness to various products offered on the market allows for enrichment of menus and daily diet, which contributes to protection against nutrient deficiencies and is important when shaping healthy eating habits. Health promotion is connected with the promotion of a healthy lifestyle, including dietary diversification and openness to new types of food. 

This study examined ‘typical’ western consumers’ readiness to adopt insects in a diet. Only 17.8% of the participants indicated to be ready to adopt insects as a food product, while another 23.1.% were undecided (Table 3). Although these numbers are not large, they indicate the willingness to try insects as a substitute for meat, at least to some extent. The willingness to eat processed insect food (bread, biscuit) was higher than for unprocessed edible insects (whole insects). This finding supports previous studies which have also shown that insects are likely to be more acceptable to western consumers when they are disguised or incorporated in familiar foods, rather than visible [16,21,34,35]. 

In accordance with the previous research [21,36,37,38], men in the present study were more likely to accept insects as food, as they tend to be more adventurous when it comes to trying new or unappealing food products. 

It is worth noting that environmental benefits were one of the least influential factors affecting the willingness to try edible insects. The vast majority of respondents (96.9%) declared that they consume animal products. In contrast, environmental benefits of the eating of insects instead of meat were found by Verbeke (2015) [26]. This author noted that attention to the environmental impact of food choice was associated with a higher likelihood of adopting insects as meat substitutes by Belgian consumers. The results of this study are consistent with the results of Wilkinson et al. (2018) [16] and indicate that people attach more importance to the sensory aspects of food (taste, appearance) than to environmental benefits.

As expected, survey results showed that respondents indicated a lower willingness to eat edible insects and insect-based products, as eating insects is not a common food practice in Poland.

Basing on the logit model, it was shown that the factors that most strongly affect the acceptance of edible insects in the students’ diet were food neophobia and travels to Asia and America (North and South), countries with different culinary traditions. The high mobility of young people suggests that their eating habits can change under the influence of other cultures and adapt to local cultures. The desire to try original dishes in specific tourist destinations is increasingly a leading motive for choosing the direction of these trips. Therefore, it can be assumed that the fashion for exotic and distant destinations observed in recent years among Polish communities will contribute to increasing acceptance of edible insects in their diet.

Many media and marketing activities are therefore needed to promote insects as an alternative food source. Education is necessary to change the way people think so that insects are perceived not as ugly, but as super food, which, in addition to its high nutritional value, has an impact on the protection of the people environment. The media should be utilized as a potential tool for nutritional education regarding the use of edible insects in human diet. Social advertising (promoting insects as a source of protein, unsaturated fatty acids), placement of food edible insect products in high visibility TV programs, and the involvement of famous people (singers, actors) in the promotion of insects in the diet are just some of the possibilities to be used in food education, which could change consumers’ attitude towards edible insects.

Perhaps it would be a good idea to give away free samples of insect-based products. This is indicated by the results of the tasting session of bread made on the basis of wheat flour blends with insect powder (Acheta domesticus) and the high interest of consumers in the new type of food. Indeed, consumer conviction that insects, and products prepared on their basis, can be tasty and look appetizing may prove to be the most effective strategy in promoting the adoption of entomophagy. House (2016) [39] suggested that if insect-based foods are to be commercially successful they will need to be at a comparable level of price, tastiness, and availability to existing western foods. 

## 5. Conclusions

Our research was aimed at getting to know the opinions on the consumption of edible insects and insect-based products among Polish communities. The results of the survey suggest that there are strong prejudices against edible insects among students, which is reflected in a low willingness to their consumption. The reluctance to try new foods is not due to a lack of knowledge, but rather a prejudice against insects as food. Our research confirmed that reducing prejudices about insects as food can be achieved by presenting insects not in a real biological form (by making them less disgusting to the sight).

The results of the study indicate that the perception of eating insect-based foods (bread, biscuit) is different from eating visible insects. Insects in invisible form, i.e., inside processed food, have a greater positive acceptance of consumption than visible insects. The introduction of insects into the diet requires a change in consumer mentality and cultural beliefs, not only in Poland, but also in other European countries, as people consider insects as “abomination”. However, when the insects are transformed, like other food products, and you cannot see them on the plate, it is easier to convince the consumer to try a new dish. The results of this research also indicate that it may be easier to accept a new taste (product) if it is served as a product already known and liked. 

Future research efforts could be directed towards examining the consumption of edible insects for other communities, as well as more in depth research on the profile of people participating in the tasting session. 

## Figures and Tables

**Table 1:** Consumer demographics characteristics.

Feature	Category		*n*	%
Sex	woman		294	64.8
	man		160	35.2
	village		125	27.5
Place of residence	city up to 20,000 inhabitants		50	11.0
	city from 20,000 to 100,000 inhabitants		90	19.8
	city from 100,000 to 500,000 inhabitants		37	8.1
	city of more than 500,000 inhabitants		152	33.5
Monthly household income per person	500.00–1000.00 PL		110	24.6
	1000.01–1500.00 PL		143	31.5
	1500.01–2000.00 PL		87	19.2
	>2000.00 PL		114	25.1
Travel destination	Europe	no	10	2.2
		yes	444	97.8
	Asia	no	407	89.6
		yes	47	10.4
	Africa	no	382	84.1
		yes	72	15.9
	South America	no	442	97.4
		yes	12	2.6
	North America	no	439	96.7
		yes	15	3.3
Protein source in the diet	beef	no	342	75.3
		yes	112	24.7
	chicken, turkey	no	39	8.6
		yes	415	91.4
	pork	no	307	67.6
		yes	147	32.4
	fish	no	256	56.4
		yes	198	43.6
	seafood	no	414	91.2
		yes	40	8.8
	none of the above	no	440	96.9
		yes	14	3.1

**Table 2:** The influence of selected variables on the food neophobia level.

Variable	Category		Food Neophobia Level ^1^						*p-*Value
			Low		Medium		High		
			*n*	%	*n*	%	*n*	%	
Sex	Total		76	16.7	294	64.8	84	18.5	0.0024 *
	woman		38	12.9	192	65.3	64	21.8	
	man		38	23.8	102	63.8	20	12.5	
Place of residence	village		21	15.8	79	59.4	33	24.8	0.8031
	city up to 20,000 inhabitants		4	7.7	37	71.2	11	21.2	
	city from 20,000 to 100,000 inhabitants		17	19.1	59	66.3	13	14.6	
	city from 100,000 to 500,000 inhabitants		6	15.0	25	62.5	9	22.5	
	city of more than 500,000 inhabitants		28	17.4	94	58.4	39	24.2	
Income^2^	500.00–1000.00 PL		12	11.7	77	74.8	14	13.6	0.2791
	1000.01–1500.00 PL		22	14.4	96	62.7	35	22.9	
	1500.01–2000.00 PL		15	17.0	56	63.6	17	19.3	
	>2000.00 PL		27	21.3	65	51.2	35	27.6	
Travel destination	Asia	no	61	15.0	267	65.6	79	19.4	0.0090 *
		yes	15	31.9	27	57.4	5	10.6	
	Africa	no	60	15.7	249	65.2	73	19.1	0.3526
		yes	16	22.2	45	62.5	11	15.3	
	North and South America	no	67	15.6	283	65.8	80	18.6	0.0185 *
		yes	9	37.5	11	45.8	4	16.7	
Protein source in the diet	beef	no	49	14.3	225	65.8	68	19.9	0.0392 *
		yes	27	24.1	69	61.6	16	14.3	
	chicken, turkey	no	5	12.8	26	66.7	8	20.5	0.7753
		yes	71	17.1	268	64.6	76	18.3	
	pork	no	45	14.7	193	62.9	69	22.5	0.0040 *
		yes	31	21.1	101	68.7	15	10.2	
	fish	no	42	16.4	164	64.1	50	19.5	0.8111
		yes	34	17.2	130	65.7	34	17.2	
	sea food	no	62	15.0	271	65.5	81	19.6	0.0025 *

^1^The resultant Food Neophobia Scale (FNS) ranged from 10.0 to 50.0 points. The obtained scores were analyzed in categories (of low, medium, or high level of food neophobia). In order to divide the respondents into FNS categories, their results were grouped as: low food neophobia level (FNS from 10.0 to 18.0 points); medium food neophobia level (FNS from 18.1 to 30.0 points); or high food neophobia level (FNS from 30.1 to 50.0 points). ^2^ Income: Monthly household income per person; *p*-values where values marked with (*) differ significantly.

**Table 3:** Distribution of answers to survey questions and *p*-value for χ^2^ tests.

Questions		Yes	No	I Don’t Know	*p*-Value		
					S ^1^	P ^2^	I ^3^
1.	Have you heard about edible insects before?	88.1	9.7	2.2	0.9199	0.5985	0.2594
2.	Have you heard of eating insects in other cultures before?	94.1	4.6	1.3	0.9773	0.1984	0.3268
3.	Have you heard about restaurants serving edible insects?	54.2	39.9	5.9	0.5791	0.0932	0.3198
4.	Do you know that edible insects are the source of proteins?	67.2	18.9	13.9	0.6916	0.5811	0.5226
5.	Have you eaten edible insects before?	7.3	90.7	2.0	0.2063	0.1367	0.0046*
6.	Is insect eating disgusting for you?	45.2	36.6	18.3	0.0000 *	0.0090 *	0.0181 *
7.	Does the idea of eating insects make you feel sick?	38.5	49.6	11.9	0.0000 *	0.4356	0.0144 *
8.	Would you accept edible insects in your diet?	17.8	59.0	23.1	0.0000 *	0.0633	0.0037 *
9.	Would you accept bread from insect flour in your diet?	31.7	47.4	20.9	0.0000 *	0.1222	0.0018 *
10.	Would you accept biscuit from insect flour in your diet?	29.7	48.2	22.0	0.0002 *	0.1108	0.0033 *
11.	Would you accept insects in animal feeding: poultry; pork; beef; fish?	62.6 60.8 61.7 63.0	23.3 23.6 22.9 22.2	14.1 15.6 15.4 14.8	0.0025 * 0.0011 * 0.0005 * 0.0257 *	0.2170 0.0187 * 0.0022 * 0.0130 *	0,7578 0.6816 0.6376 0.6807
12.	Would you like to taste whole insects served sauté?	16.1	68.9	15.0	0.0001 *	0.0182 *	*0.*0098 *
13.	Would you like to try whole insects with the addition of: spices, covered with chocolate?	35,0 24.7	54.2 61.7	10.8 13.7	0.0009 * 0.0002 *	0.0348 * 0.1521	0.0151 * 0.0131 *
14.	Would you like to try insects in an invisible form - flour added to: biscuit, bread, burger, paté?	38.8 42.5 41.0 29.5	47.1 43.6 45.6 57.0	14.1 13.9 13.4 13.4	0.0098 * 0.0133 * 0.0082 * 0.0227 *	0.3378 0.2331 0.4921 0.9055	0.0579 0.0435 * 0.0118 * 0.0092 *
15.	Would you use edible insects if you knew that: insects are easy to prepare, do not require long heat treatment, they can be bought near the place of residence, they are easily available in stores and supermarkets?	20.5 19.4 24.0 25.3	55.7 58.4 54.8 52.4	23.8 22.2 21.1 22.2	0.0000 * 0.0000 * 0.0000 * 0.0000 *	0.0675 0.3166 0.1030 0.1098	0.0399 * 0.0139 * 0.0453 * 0.0209 *
16.	Should insects be included in Western diets to solve problems with: nutrition, food security, environmental sustainability, reduced food wastage, scarcity of agricultural land, animal welfare?	34.1 22.0 29.5 37.7 35.0 33.5	27.8 33.9 31.9 24.7 25.6 27.8	38.1 44.1 38.5 37.7 39.4 38.8	0.2853 0.0096 * 0.0012 * 0.0002 * 0.0044 * 0.0834	0.7264 0.7076 0.8329 0.8405 0.5944 0.6098	0.7958 0.4862 0.2074 0.1397 0.1397 0.2093

S ^1^, sex; P ^2^, place of residence; I ^3^, monthly income; *p*-values, where values marked with (*) differ significantly.

**Table 4:** Factors influencing students’ willingness to try edible insects.

Factors	Total Score
Taste	1018
Quality	1015
Appearance	799
Food safety	704
High nutritional value	560
Price	289
Environmental benefits	164

**Table 5:** Estimation results of the logit model of the acceptance of edible insects (IA) in the students’ diet.

Variable	Parameter β	*p*-Value	Odds Ratio
Constant	–2.821	<0.001	
Sex (S)	0.653	0.017	1.922
Food neophobia level (F)	1.452	<0.001	4.270
Place of residence (PR)	0.648	0.032	1.911
Travel to Asia (TA_z_)	0.972	0.009	2.644
Travel to North and South America (TA_m_)	1.323	0.008	3.753
Goodness of fit statistics: Likelihood ratio (5) = 66.26; *p* < 0.001 Hosmer–Lemeshow = 1.339; *p* = 0.72 % correct predictions = 84.4% Nagelkerke’s R^2^ = 0.223			

## Supplementary Materials

The following are available online at https://www.mdpi.com/1660-4601/17/7/2427/s1, Table S1: Distribution of answers to survey questions by sex, place of residence, and income in case of statistically significant dependences.
