# Supplementary material for: Consumers’ Attitudes Facing Entomophagy: Polish Case Perspectives
Source: Int J Environ Res Public Health. 2020 Apr 2;17(7):2427. doi: 10.3390/ijerph17072427 (PMC7177372; doi:10.3390/ijerph17072427)
Supplement: Supplementary file 1 [file ijerph-17-02427-s001.pdf]

Table S1. Distribution of answers to survey questions by sex, place of residence and income in case of statistically significant dependences.

Variable	Category	Yes		No		I don`t know	
		Have you eaten edible insects before? (question 5)					
		n	%	n	%	n	%
Income	500.00–1000.00 PL	5	4.55	105	95.45	0	0.00
	1000.01–1500.00 PL	6	4.20	132	92.31	5	3.50
	1500.01–2000.00 PL	5	5.75	79	90.80	3	3.45
	>2000.00 PL	17	14.91	96	84.21	1	0.88
		Is insect eating disgusting for you? (question 6)					
Sex	woman	154	52.38	85	28.91	55	18.71
	man	51	31.88	81	50.63	28	17.50
Place of residence	village	59	47.20	38	30.40	28	22.40
	city up to 20,000 inhabitants	29	58.00	10	20.00	11	22.00
	city from 20,000 to 100,000 inhabitants	37	41.11	40	44.44	13	14.44
	city from 100,000 to 500,000 inhabitants	23	62.16	11	29.73	3	8.11
	city of more than 500,000 inhabitants	57	37.50	67	44.08	28	18.42
Income	500.00–1000.00 PL	62	56.36	28	25.45	20	18.18
	1000.01–1500.00 PL	70	48.95	50	34.97	23	16.08
	1500.01–2000.00 PL	35	40.23	36	41.38	16	18.39
	>2000.00 PL	38	33.33	52	45.61	24	21.05
		Does the idea of eating insects make you feel sick? (question 7)					
Sex	woman	135	45.92	122	41.50	37	12.59
	man	40	25.00	103	64.38	17	10.63
Income	500.00–1000.00 PL	51	46.36	41	37.27	18	16.36
	1000.01–1500.00 PL	58	40.56	69	48.25	16	11.19
	1500.01–2000.00 PL	34	39.08	48	55.17	5	5.75
	>2000.00 PL	32	28.07	67	58.77	15	13.16
		Would you accept edible insects in your diet? (question 8)					
Sex	woman	39	13.27	198	67.35	57	19.39
	man	42	26.25	70	43.75	48	30.00
Income	500.00–1000.00 PL	11	10.00	81	73.64	18	16.36
	1000.01–1500.00 PL	24	16.78	84	58.74	35	24.48
	1500.01–2000.00 PL	22	25.29	48	55.17	17	19.54
	>2000.00 PL	24	21.05	55	48.25	35	30.70
		Would you accept bread from insect flour in your diet? (question 9)					
Sex	woman	72	24.49	159	54.08	63	21.43
	man	72	45.00	56	35.00	32	20.00
Income	500.00–1000.00 PL	27	24.55	70	63.64	13	11.82
	1000.01–1500.00 PL	41	28.67	65	45.45	37	25.87
	1500.01–2000.00 PL	31	35.63	33	37.93	23	26.44

	>2000.00 PL	45	39.47	47	41.23	22	19.30
		Would you accept in your diet biscuit from insect flour? (question 10)					
Sex	woman	69	23.47	160	54.42	65	22.11
	man	66	41.25	59	36.88	35	21.88
Income	500.00–1000.00 PL	25	22.73	70	63.64	15	13.64
	1000.01–1500.00 PL	38	26.57	64	44.76	41	28.67
	1500.01–2000.00 PL	31	35.63	34	39.08	22	25.29
	>2000.00 PL	41	35.96	51	44.74	22	19.30
		Would you accept insects in animal feeding (poultry)? (question 11)					
Sex	woman	169	57.48	83	28.23	42	14.29
	man	115	71.88	23	14.38	22	13.75
		Would you accept insects in animal feeding (pork)? (question 11)					
Sex	woman	164	55.78	85	28.91	45	15.31
	man	112	70.00	22	13.75	26	16.25
Place of residence	village	75	60.00	32	25.60	18	14.40
	city up to 20,000 inhabitants	26	52.00	15	30.00	9	18.00
	city from 20,000 to 100,000 inhabitants	53	58.89	26	28.89	11	12.22
	city from 100,000 to 500,000 inhabitants	18	48.65	6	16.22	13	35.14
	city of more than 500,000 inhabitants	104	68.42	28	18.42	20	13.16
		Would you accept insects in animal feeding (beef)? (question 11)					
Sex	woman	164	55.78	83	28.23	47	15.99
	man	116	72.50	21	13.13	23	14.38
Place of residence	village	75	60.00	32	25.60	18	14.40
	city up to 20,000 inhabitants	25	50.00	17	34.00	8	16.00
	city from 20,000 to 100,000 inhabitants	58	64.44	23	25.56	9	10.00
	city from 100,000 to 500,000 inhabitants	18	48.65	5	13.51	14	37.84
	city of more than 500,000 inhabitants	104	68.42	27	17.76	21	13.82
		Would you accept insects in animal feeding (fish)? (question 11)					
Sex	woman	173	58.84	76	25.85	45	15.31
	man	113	70.63	25	15.63	22	13.75
Place of residence	village	76	60.80	30	24.00	19	15.20
	city up to 20,000 inhabitants	27	54.00	14	28.00	9	18.00
	city from 20,000 to 100,000 inhabitants	58	64.44	25	27.78	7	7.78
	city from 100,000 to 500,000 inhabitants	18	48.65	7	18.92	12	32.43
	city of more than 500,000 inhabitants	107	70.39	25	16.45	20	13.16
		Would you like to taste whole insects served sauté? (question 12)					

Sex	woman	31	10.54	219	74.49	44	14.97
	man	42	26.25	94	58.75	24	15.00
Place of residence	village	12	9.60	96	76.80	17	13.60
	city up to 20,000 inhabitants	5	10.00	40	80.00	5	10.00
	city from 20,000 to 100,000 inhabitants	18	20.00	62	68.89	10	11.11
	city from 100,000 to 500,000 inhabitants	4	10.81	22	59.46	11	29.73
	city of more than 500,000 inhabitants	34	22.37	93	61.18	25	16.45
Income	500.00–1000.00 PL	8	7.27	87	79.09	15	13.64
	1000.01–1500.00 PL	18	12.59	102	71.33	23	16.08
	1500.01–2000.00 PL	20	22.99	56	64.37	11	12.64
	>2000.00 PL	27	23.68	68	59.65	19	16.67
		Would you like to try whole insects with the addition of spices (question 13)					
Sex	woman	85	28.91	176	59.86	33	11.22
	man	74	46.25	70	43.75	16	10.00
Place of residence	village	35	28.00	75	60.00	15	12.00
	city up to 20,000 inhabitants	12	24.00	32	64.00	6	12.00
	city from 20,000 to 100,000 inhabitants	26	28.89	54	60.00	10	11.11
	city from 100,000 to 500,000 inhabitants	15	40.54	18	48.65	4	10.81
	city of more than 500,000 inhabitants	71	46.71	67	44.08	14	9.21
Income	500.00–1000.00 PL	27	24.55	73	66.36	10	9.09
	1000.01–1500.00 PL	47	32.87	77	53.85	19	13.29
	1500.01–2000.00 PL	36	41.38	46	52.87	5	5.75
	>2000.00 PL	49	42.98	50	43.86	15	13.16
		Would you like to try whole insects covered with chocolate? (question 13)					
Sex	woman	58	19.73	202	68.71	34	11.56
	man	54	33.75	78	48.75	28	17.50
Income	500.00–1000.00 PL	16	14.55	81	73.64	13	11.82
	1000.01–1500.00 PL	36	25.17	82	57.34	25	17.48
	1500.01–2000.00 PL	23	26.44	57	65.52	7	8.05
	>2000.00 PL	37	32.46	60	52.63	17	14.91
		Would you like to try insects in an invisible form—flour added to biscuit? (question 14)					
Sex	woman	102	34.69	154	52.38	38	12.93
	man	74	46.25	60	37.50	26	16.25
		Would you like to try insects in an invisible form—flour added to bread? (question 14)					
Sex	woman	111	37.76	142	48.30	41	13.95
	man	82	51.25	56	35.00	22	13.75
Income	500.00–1000.00 PL	42	38.18	59	53.64	9	8.18
	1000.01–1500.00 PL	53	37.06	63	44.06	27	18.88
	1500.01–2000.00 PL	41	47.13	35	40.23	11	12.64
	>2000.00 PL	57	50.00	41	35.96	16	14.04

		Would you like to try insects in an invisible form—flour added to burger? (question 14)					
Sex	woman	105	35.71	145	49.32	44	14.97
	man	81	50.63	62	38.75	17	10.63
Income	500.00–1000.00 PL	35	31.82	62	56.36	13	11.82
	1000.01–1500.00 PL	56	39.16	59	41.26	28	19.58
	1500.01–2000.00 PL	39	44.83	42	48.28	6	6.90
	>2000.00 PL	56	49.12	44	38.60	14	12.28
		Would you like to try insects in an invisible form—flour added to paté? (question 14)					
Sex	woman	74	25.17	178	60.54	42	14.29
	man	60	37.50	81	50.63	19	11.88
Income	500.00–1000.00 PL	28	25.45	73	66.36	9	8.18
	1000.01–1500.00 PL	36	25.17	76	53.15	31	21.68
	1500.01–2000.00 PL	29	33.33	50	57.47	8	9.20
	>2000.00 PL	41	35.96	60	52.63	13	11.40
		Would you use edible insects if you knew that: insects are easy to prepare? (question 15)					
Sex	woman	45	15.31	186	63.27	63	21.43
	man	48	30.00	67	41.88	45	28.13
Income	500.00–1000.00 PL	17	15.45	75	68.18	18	16.36
	1000.01–1500.00 PL	25	17.48	78	54.55	40	27.97
	1500.01–2000.00 PL	23	26.44	45	51.72	19	21.84
	>2000.00 PL	28	24.56	55	48.25	31	27.19
		Would you use edible insects if you knew that: do not require long heat treatment? (question 15)					
Sex	woman	41	13.95	195	66.33	58	19.73
	man	47	29.38	70	43.75	43	26.88
Income	500.00–1000.00 PL	17	15.45	78	70.91	15	13.64
	1000.01–1500.00 PL	22	15.38	79	55.24	42	29.37
	1500.01–2000.00 PL	23	26.44	45	51.72	19	21.84
	>2000.00 PL	26	22.81	63	55.26	25	21.93
		Would you use edible insects if you knew that: they can be bought near the place of residence? (question 15)					
Sex	woman	53	18.03	185	62.93	56	19.05
	man	56	35.00	64	40.00	40	25.00
Income	500.00–1000.00 PL	14	14.29	69	70.41	15	15.31
	1000.01–1500.00 PL	29	20.28	76	53.15	38	26.57
	1500.01–2000.00 PL	26	29.89	45	51.72	16	18.39
	>2000.00 PL	33	28.95	55	48.25	26	22.81
		Would you use edible insects if you knew that: they are easily available in stores and supermarkets? (question 15)					
Sex	woman	56	19.05	177	60.20	61	20.75
	man	59	36.88	61	38.13	40	25.00

Income	500.00–1000.00 PL	24	21.82	69	62.73	17	15.45
	1000.01–1500.00 PL	27	18.88	76	53.15	40	27.97
	1500.01–2000.00 PL	30	34.48	39	44.83	18	20.69
	>2000.00 PL	34	29.82	54	47.37	26	22.81
		Should insects be included in Western diets to solve problems with: food security? (question 16)					
Sex	woman	52	17.69	107	36.39	135	45.92
	man	48	30.00	47	29.38	65	40.63
		Should insects be included in Western diets to solve problems with: environmental sustainability? (question 16)					
Sex	woman	71	24.15	107	36.39	116	39.46
	man	63	39.38	38	23.75	59	36.88
		Should insects be included in Western diets to solve problems with: reduced food wastage? (question 16)					
Sex	woman	91	30.95	84	28.57	119	40.48
	man	80	50.00	28	17.50	52	32.50
		Should insects be included in Western diets to solve problems with: scarcity of agricultural land? (question 16)					
Sex	woman	87	29.59	82	27.89	125	42.52
	man	72	45.00	34	21.25	54	33.75

Data are presented as: n (number of respondents) and % (percentages).
